# Myrrh and Chamomile Flower Extract Inhibit Mediator Release from IgE-stimulated Mast-Cell-Like RBL-2H3 Cells

**DOI:** 10.3390/plants11243422

**Published:** 2022-12-08

**Authors:** Fabian Altenbernd, Lena Schwarz, Bartosz Lipowicz, Cica Vissiennon

**Affiliations:** 1 Institute of Medical Physics and Biophysics, Medical Faculty, Leipzig University, Härtelstr. 16-18, 04107 Leipzig, Germany; 2 Repha GmbH Biologische Arzneimittel, Alt-Godshorn 87, 30855 Langenhagen, Germany

**Keywords:** Myrrh, chamomile flower, IBS, chronic disease, natural compounds, mast cells, RBL-2H3

## Abstract

Recent clinical evidence supports the efficacy of a traditional medicinal product (TMP) containing a combination of myrrh (*Commiphora myrrha* (Nees) Engl.), coffee charcoal (*Coffea arabica* L.), and chamomile flower dry extract (*Matricaria chamomilla* L.) in the therapy of diarrhea and inflammatory bowel disease. Mast cells seem to play a key role in the symptom generation of irritable bowel syndrome (IBS). To evaluate the use of the TMP in IBS treatment, the effects of the herbal extracts on the release of mast-cell mediators from stimulated RBL-2H3 cells were investigated. Therefore, degranulation was induced by phorbol-12-myristate-13-acetate (PMA) and calcium ionophore A13187 (CI) or IgE stimulation, and the amounts of released β-hexosaminidase and histamine were quantified. The extracts showed no effect on the mediator release of PMA- and CI-stimulated RBL-2H3 cells. Myrrh and chamomile were able to reduce the β-hexosaminidase release of IgE-stimulated cells, while myrrh showed stronger inhibition of the mediator release than chamomile, which reduced only IgE-stimulated histamine release. Thus, these results indicate a mechanistic basis for the use of the herbal combination of myrrh, coffee charcoal, and chamomile flower extract in the symptom-oriented treatment of IBS patients with diarrheal symptoms.

## 1. Introduction

Irritable bowel syndrome (IBS) affects about 11% of the global population [1]. Patients suffer a huge variety of symptoms such as pain, discomfort, diarrhea, obstipation, and flatulence, leading to a decrease in their quality of life [2]. While the etiology and pathophysiology of the disease have yet to be fully unraveled, the increased amount and activity of mast cells in IBS patients might play a key role in symptom generation [3,4]. Upon degranulation, mediators such as histamine and proteases are released, activating corresponding receptors on the surface of epithelial cells, which leads to chloride secretion into the gut lumen causing diarrhea [5,6,7,8]. Proteases and protease-activated receptors (PARs) are associated with the impairment of intestinal barrier function [8,9,10]. Furthermore, mast cell mediators such as histamine and tryptase are able to excite visceral nerves [11,12,13], possibly explaining the dysfunction in motility, discomfort, and pain. Therefore, inhibiting the mediator release from mast cells should improve symptoms in IBS patients. This effect could be observed in a randomized, placebo-controlled study with ketotifen, which significantly improved symptoms associated with IBS such as diarrhea, discomfort, and pain, therefore improving the overall quality of life in patients with diarrhea-predominant IBS. It was also shown that ketotifen decreased the spontaneous mediator release from mast cells [14,15].

In severe IBS cases, patients cannot fulfil their daily tasks or participate in social activities, which drastically decreases their quality of life [2,16]. According to the German S3 guideline, IBS treatment is only symptomatic [17]. Due to the lack of satisfying treatment options to fully control symptoms, patients’ interest in complementary medicine (CM) has increased. However, because of scarce evidence, CM has a low acceptance rate by healthcare professionals. Further pharmacological validation of CM might contribute to higher acceptance and rational use of CM and therefore increase the quality of life of IBS patients. Myrrhinil-Intest^®^ is a traditional herbal medicinal product containing a combination of myrrh (*Commiphora myrrha* (Nees) Engl.), coffee charcoal (*Coffea arabica* L.), and chamomile flower dry extract (*Matricaria chamomilla* L.), which is used for the treatment of diarrhea and gut disorders. Clinical efficacy and safety have been shown in diarrhea and IBS patients [18].

Previous pharmacological in vitro studies demonstrated spasmolytic, anti-inflammatory, and intestinal-barrier-stabilizing effects of the plant compounds [19,20,21,22]. To further evaluate the pharmacological profile of the herbal combination product in the treatment of IBS, the aim of this study was to investigate the influence of the herbal components on mast-cell degranulation. Thus, characterized extracts were applied in a mast-cell degranulation model using RBL-2H3 cells, and the mediators released (β-hexosaminidase, histamine) were investigated after chemical as well as IgE stimulation.

## 2. Results and Discussion

To support the hypothesis that the antidiarrheal effects of the herbal combination might be related to the compounds inhibiting the mediator release from mucosal mast cells, the potential effects were investigated using the cell line RBL-2H3. The cell line can be used as a model for mucosal mast cells, releasing different mediators upon degranulation, induced by a variety of compounds as well as IgE sensitization followed by antigen stimulation [23,24,25]. Degranulation was induced by chemical agents, phorbol-12-myristate-13-acetate (PMA) and calcium ionophore A13187 (CI), or by IgE stimulation. The protease β-hexosaminidase and histamine are preformed mediators stored in RBL-2H3 cell granules and were therefore chosen as degranulation markers.

### 2.1. Plant Extract Characterization

All extracts used in the following in vitro studies have been previously characterized, mainly via liquid chromatography and subsequent mass spectrometry [20,21,22].

HPLC-DAD analysis of the chamomile extract revealed seven main peaks [20]. UV-spectra for five compounds (Table 1, peaks 3 to 7) showed typical UV spectra of flavonoids. Comparison with reference standards as well as mass spectrometry confirmed apigenin and apigenin derivatives as the main constituents of the extract (Table 1, peaks 3 to 7). Peaks 1 and 2 (Table 1) were identified as derivatives of ferulic acid.

Mass spectrometry alongside 1D and 2D NMR of isolated compounds from the myrrh extract showed the presence of several sesquiterpenes (Table 2, peaks 1 to 6) and one (nor-)triterpenoid (Table 3, peak 7) [21]. Comparison of the NMR and mass spectrometry data with literature allowed for the identification of all seven substances.

Analysis of the coffee charcoal extract via HPLC, reference standards, and literature data led to the identification of caffeine (Table 3 peak 4) and trigonelline (Table 2, peak 1) as well as several caffeic acid derivatives (Table 2, peaks 2, 3, and 5) and two feruloylquinic acid isomers (Table 2, peaks 6 and 7) [22].

### 2.2. Influence on Cell viability

**Figure 1:**
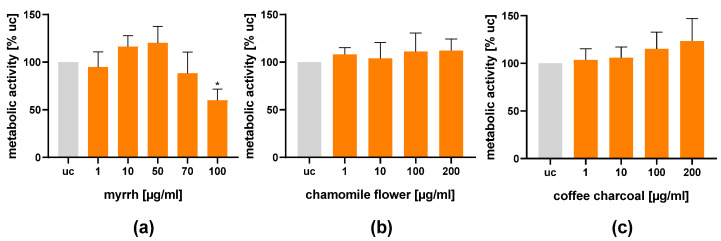
Effects of the plant extracts on the metabolic activity of RBL-2H3 cells. Cells were incubated overnight (18 h) with (**a**) myrrh resin extract (1–100 µg/mL); (**b**) chamomile flower extract (1–200 µg/mL); (**c**) coffee charcoal extract (1–200 µg/mL) and incubated for 2 h with XTT-assay solution. Results are presented as means ± SEM; *n* = 4 (myrrh); *n* = 6; * *p* < 0.05.

Before conducting any experiments, the extracts were tested for cytotoxicity using an XTT assay (Figure 1). Only myrrh at a concentration of 100 µg/mL reduced the metabolic activity of the RBL-2H3 cells (59.9% ± 10.2%; *p* < 0.05). Based on these results, concentrations were chosen for further experiments.

### 2.3. Influence on Chemical Induced Mediator Release

**Figure 2:**
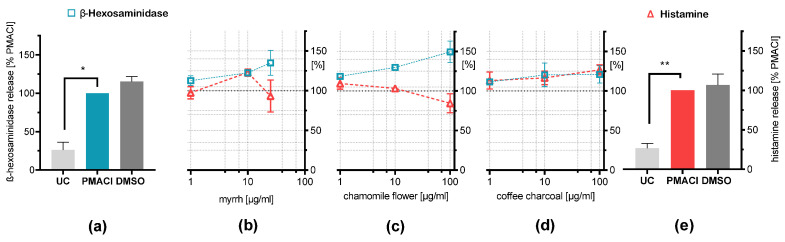
Effects of the plant extracts on mediator release of PMA and CI stimulated RBL-2H3 cells. β-hexosaminidase (□) and histamine (∆) release were determined after 30 min stimulation with 40 nM PMA + 1 µM CI (PMACI = 100%) compared untreated control (UC) and vehicle control (PMACI + 0.2% DMSO) (**a**,**e**). (**b**) Myrrh resin extract (1–50 µg/mL), (**c**) chamomile flower extract (1–200 µg/mL), or (**d**) coffee charcoal extract (1–200 µg/mL) were incubated overnight (18 h) before PMACI stimulation. Results are presented as means ± SEM. *n* = 3; * *p* < 0.05; ** *p* < 0.01.

Upon stimulation with a combination of CI (1 µM) and PMA (40 nM), the plant extracts were unable to exhibit an effect on the release of β-hexosaminidase and histamine from RBL-2H3 cells compared to the stimulated control (Figure 2). Since the degranulation is induced by increasing intracellular Ca^2+^ concentration through CI, as a pore-forming agent or carrier [25], this method resembles a non-physiological mechanism. Therefore, experiments using a stimulation method closer to physiological conditions were performed to investigate the potential inhibitory effects of the plant extracts.

### 2.4. Influence on IgE-Induced β-Hexosaminidase Release

**Figure 3:**
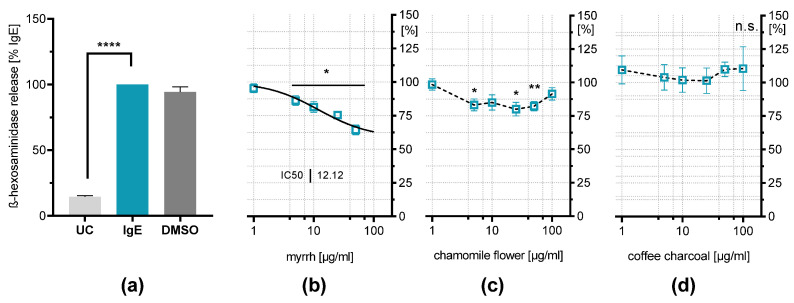
Effects of the plant extracts on β-hexosaminidase release of IgE-stimulated RBL-2H3 cells. β-hexosaminidase release was determined after overnight (18 h) incubation with 500 ng/mL IgE following 30 min stimulation with 1 µg/mL DNP-HSA (IgE = 100%) compared untreated control (UC) and vehicle control (IgE + 0.2% DMSO) (**a**). (**b**) Myrrh resin extract (1–50 µg/mL), (**c**) chamomile flower extract (1–100 µg/mL) or (**d**) coffee charcoal extract (1–100 µg/mL) were incubated simultaneously to IgE. Results are presented as means ± SEM and non-linear regression for myrrh (continuous line); *n* = 4–10; * *p* < 0.05; ** *p* < 0.01; **** *p* < 0.0001; n.s. non-significant.

RBL-2H3 cell degranulation can be induced by IgE stimulation. The binding of antigens by surface-bound IgE molecules leads to FcεR-activation, resulting in degranulation of the cells [24], which resembles a more physiological pathway of mast cell degranulation. It is shown that IgE-stimulated β-hexosaminidase release from RBL-2H3 was reduced by myrrh resin extract down to 64.3% (±3.5%, *p* < 0.05) and chamomile flower extract down to 80.4% (±5.0%, *p* < 0.05) (Figure 3). The effect of chamomile flower extract aligns with findings showing flavonoids are able to reduce mediator release from RBL-2H3 [26,27]. Apigenin has shown inhibitory effects on β-hexosaminidase release upon IgE stimulation, which is also present in the extract [28,29]. These results indicate a U-shaped response curve. This is commonly observed for phytochemical compounds, where they exhibit positive effects at low concentrations, while the opposite effect can be found at higher concentrations, which is called hormesis [30,31,32].

Myrrh resin extract shows a concentration-dependent inhibitory effect on IgE-induced β-hexosaminidase release, reducing it by about one-third compared to the stimulated control at the highest concentration of 50 µg/mL (64.3% ± 4.9%; *p* < 0.0001). IC_50_ is reached at a concentration of 12.12 µg/mL. This result suggests inhibitory properties from mast-cell mediator release upon IgE stimulation, which is underlined by the findings of a study with HMC-1 cells showing *Commiphora myrrha* (Nees) Engl. extract reducing histamine release and IL-31 production [33]. The coffee charcoal extract had no effect on the mediator release.

### 2.5. Influence on IgE-Induced Histamine Release

**Figure 4:**
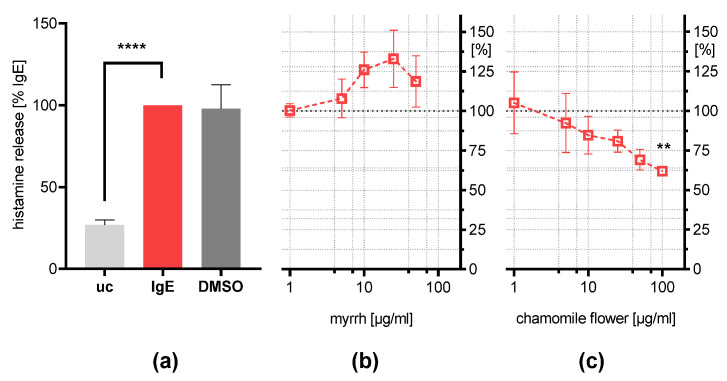
Effects of the plant extracts on the histamine release of IgE-stimulated RBL-2H3 cells. Histamine release was determined after overnight (18 h) incubation with IgE (500 ng/mL) following 30 min stimulation with 1 µg/mL DNP-HSA (IgE = 100%) compared to the untreated control (UC) and vehicle control (IgE + 0.2% DMSO) (**a**). (**b**) Myrrh resin extract (1–50 µg/mL), or (**c**) chamomile flower extract (1–100 µg/mL) were incubated simultaneously to IgE. Results are presented as means ± SEM; *n* = 5; ** *p* < 0.01; **** *p* < 0.0001.

For further investigation of these results, the histamine concentration was determined in the supernatant of IgE/HSA-stimulated cells treated with myrrh and chamomile flower extract (Figure 4). Contrary to the effect observed for β-hexosaminidase release, only the chamomile flower, at a concentration of 100 µg/mL, was able to reduce the histamine release (61.9% ± 6.9%; *p* < 0.01). The inhibition might be induced by flavonoids such as apigenin and luteolin, as previous studies suggested [26,28,34].

These results indicate that the inhibition of β-hexosaminidase release is independent of the amount of histamine released in IgE-stimulated RBL-2H3 cells. Studies on mice bone marrow mast cells showed that they form various secretory granules, which all store β-hexosaminidase; however, some do not contain histamine [35,36]. Furthermore, mast cells are capable of releasing β-hexosaminidase independently of histamine [36,37]. This might serve as a possible explanation as to why myrrh reduces β-hexosaminidase release without inhibiting histamine secretion. This heterogeneity of mast cell granules could not be observed in human mast cells in vivo [38], which suggests that myrrh resin might be able to inhibit the release of all mediators of mucosal mast cells. This would include PAR-2 agonist tryptase, which could explain the antidiarrheal activity in IBS since PAR-2 activation plays a role in symptom generation [8,9,10,39]. The herbal extracts also exhibit anti-inflammatory and barrier-stabilizing effects on epithelial cells [21,40]. Further studies could investigate the barrier-stabilizing effects of the extracts on the epithelial cells in the presence of PAR-2 agonists, or stimulated mast cells in a co-culture model. Combined with the inhibition of histamine release due to flavonoids in chamomile flower extract, these results indicate multimodal effects, which may underly the symptom reduction observed in IBS patients and further validates the use of a TMP in IBS therapy.

## 3. Materials and Methods

### 3.1. Chemicals

Ethanol for extraction was purchased in HSL quality from CSC Jäckelchemie (Rauschwitz, Germany) and purified by evaporation. Dimethyl sulfoxide (DMSO) for solving plant extracts was purchased from VWR (Radnor, PA, USA). Chemicals (p.a. grade) were supplied by Merck (Darmstadt, Germany; glucose, CaCl_2_·2H_2_O; MgCl·2H_2_O; citric acid monohydrate), Carl Roth (Karlsruhe, Germany; HEPES), Fluka (Buchs, Switzerland; KCl, Triton-X), Th. Geyer (Renningen, Germany; NaCl), or Sigma (St. Louis, MO, USA; NaHPO_4_·2H_2_O; glycine, bovine serum albumin (BSA); phorbol-12-myristate-13-acetate (PMA); calcium ionophore A13187 (CI); di-nitrophenyl human serum albumin (DNP-HSA); mouse anti-DNP-HSA-IgE; 4-nitrophenyl-n-acetyl-ß-d-glucosaminide (pNAG)). Cell culture supplies MEM, fetal bovine serum (FBS), penicillin-streptomycin (P/S), glutamine stable, trypsin-EDTA and Dulbecco’s phosphate-buffered saline (DPBS) were provided by Biowest (Riverside, MO, USA).

### 3.2. Cell Culture

RBL-2H3 cells, purchased from DMSZ (Braunschweig, Germany), were cultured with MEM supplemented with 10% FBS, 1% P/S, and 1% glutamine stable. All cell lines were cultivated under standard cell-culture conditions. For chemical stimulation, RBL-2H3 cells were seeded into a sterile 96-well plate (GBO (Frickenhausen, Germany)) at 1 × 10^5^ cells/mL density, grown overnight, washed with DPBS, incubated for 30 min with plant extracts and control solutions, washed again and incubated for 30 min with 100 µL of a freshly prepared solution of 40 nM PMA and 1 µM CI in HEPES-buffer (HEPES 10 mM, NaCl 137 mM, KCl 2.7 mM, Na_2_HPO_4_·2H_2_O 0.4 mM, glucose 5.6 mM, CaCl_2_·2H_2_O 1.8 mM, MgCl·2H_2_O 1.3 mM, BSA 0,4%). For IgE stimulation, 5 × 10^5^ cells/mL were seeded into 96-well plates, before plant extracts and 500 ng/mL anti-DNP-HSA-IgE were added and incubated overnight (18 h) for sensitization. Cells were washed with HEPES buffer and incubated with 100 µL of 1 µg/mL DNP-HSA solution in HEPES for 30 min.

### 3.3. Plant Material

Ready-to-use chamomile flower dry extract (EtOH 60% *m*/*m*; DER: 4–6:1, batch-no. HC0070) as well as powdered myrrh (Myrrhe gum EB/PB pulv., batch-no. NM0160) and powdered coffee charcoal (*Carbo Coffea* EB 6, batch-no. JB0142) were provided by Lomapharm (Emmerthal, Germany).

Myrrh and coffee charcoal extracts were prepared, lyophilized, and stored as previously described by Weber et al. [21,22].

### 3.4. Quantification of β-Hexosaminidase Release

The β-hexosaminidase release was analyzed similarly to Kuehn et al. [41]. Briefly, 50 µL of the supernatants or lysed cells (addition of 150 µL of Triton-X 0.1%) was transferred into a 96-well plate (GBO (Frickenhausen, Germany)) and incubated with 100 µL of pNAG-solution (3.5 mg/mL in citrate buffer (citric acid monohydrate 40 nM, Na_2_HPO_4_·2H_2_O 20 mM) pH = 4.5) for 90 min at 37 °C without CO_2_. The enzymatic reaction was stopped by the addition of 50 µL glycine buffer pH = 10.7 (glycine 400 nM). Extinction was measured at 405 nm (reference 620 nm) with an Infinite^®^ M200 plate reader (TECAN, Salzburg, Austria).

### 3.5. Quantification of Histamine Release

The histamine concentration in the supernatants was determined using the Histamine ELISA kit (cat no A05890), provided by Bertin (Montigny-le-Bretonneux, France), according to the manufacturer’s instructions.

### 3.6. Influence on Cell Viability

The measurement of the metabolic activity for the RBL-2H3 cells after overnight incubation in the presence of the plant extract was performed with the XTT-Vitality-Assay Kit provided by Roche (Basel, Switzerland), according to the manufacturer’s instructions.

### 3.7. Data Analysis

Microsoft Excel (Office 365) was used for data capturing and processing of the extinction measurements. For statistical evaluation, a mixed-model one-way analysis of variance (ANOVA) followed by Dunnett’s multiple comparisons test against the stimulated control was conducted with GraphPad Prism 8.0.2 (* *p* < 0.05, ** *p* < 0.01, *** *p* < 0.001, **** *p* < 0.0001.

## 4. Conclusions

The RBL-2H3 cell line was used as a model for mucosal mast cells to investigate the effects of myrrh, coffee charcoal, and chamomile flower extracts on degranulation. In IgE-stimulated cells, myrrh and chamomile flower extracts were able to inhibit the release of the protease β-hexosaminidase, while chamomile flower extract exhibited a moderate effect on histamine release, probably driven by flavonoids.

In this context and with the available clinical evidence suggesting the antidiarrheal properties of the herbal combination of myrrh, coffee charcoal, and chamomile flower, this study indicates a potential target on mucosal mast cells and builds a foundation for further investigation into the mechanism of action, as well as supporting its rational use in IBS. It also underlines the multi-target approach of herbal treatments in complex diseases such as IBS, to improve the quality of life of patients.

## Figures and Tables

**Table 1:** Summary of the chromatographic, UV-vis-spectroscopic and mass spectrometric characteristics of the plant compounds identified in chamomile flower extract. [20].

Peak	RT (min)	Peak Height (mAU)	Molecular Weight	Compound
1	42.182	73.6	73.6	(Iso)ferulic acid O-glucoside
2	50.317	163.4	163.4	(Iso)ferulic acid O-glucosyl ester
3	57.433	524.6	524.6	Apigenin-7-O-Glucoside
4	60.533	65.0	65.0	Apigenin-glucosyl-monoacetate
5	62.467	84.4	84.4	Apigenin-7-O-malonylglucoside
6	66.317	149.9	149.9	Apigenin-glucosyl-monoacetate
7	70.483	49.7	49.7	Apigenin

**Table 2:** Summary of the chromatographic, UV-vis-spectroscopic and mass spectrometric characteristics of the plant compounds identified in coffee charcoal extracts [22].

Peak	RT (min)	λ max (nm)	*m*/*z*	Ion	Compound
1	4.1	200, 263	138.12	[M + H]^+^	Trigonelline
2	32.778	244, 325	352.96	[M − H]^−^	Neochlorogenic acid (3-caffeoylquinic acid)
			706.84	[2M − H]^−^	
3	39.743	244, 325	190.92	[quinic acid − H]^−^	Chlorogenic acid (5-caffeoylquinic acid)
			352.98	[M − H]^−^	
			707.13	[2M − H]^−^	
4	40.093	218, 272	138.12	[M + H − OCNCH_3_]^+^	Caffeine
			163.08	[M + H − CH_3_OH]^+^	
			195.08	[M + H]^+^	
5	40.646	244, 325	190.95	[quinic acid − H]^−^	Cryptochlorogenic acid (4-caffeoylquinic acid)
			352.96	[M − H]^−^	
			707.13	[2M − H]^−^	
6	44.807	238	172.95	[quinic acid − H − H_2_O]^−^	Feruloylquinic acid
			192.96	[ferulic acid − H]^−^	
			366.99	[M − H]^−^	
			734.89	[2M − H]^−^	
7	46.266	235	172.93	[quinic acid – H − H_2_O]^−^	Feruloylquinic acid
			190.92	[quinic acid − H]^−^	
			366.98	[M − H]^−^	
			735.15	[2M − H]^−^	

**Table 3:** Summary of LC-/GC-MS characteristics of plant compounds identified in myrrh extract. Retention times (RT in min) and mass spectra (m/z) for furanoeudesma-1,3-diene (1), curzerenone (2), 2-methoxy-5-acetoxyfuranogermacr-1(10)-en-6-one (3), 5-αH,8-βH-eudesma-1,3,7(11)-trien-8,12-olide (4), hydroxylindestrenolide (5), hydroxyisogermafurenolide (6), and 3,4-secomansumbinoic acid (7) were determined in the standard solutions and the myrrh extract. Analyses were performed either on LC (^a^) or GC (^b^) in positive (^c^) or negative (^d^) mode. [21].

Standard	RT (Standard)	*m*/*z* (Standard)	RT (Extract)	*m/z* (Extract)	Molecular Formula
1	6.11 ^a^	214.1345 ^c^	6.11 ^a^	214.1347 ^c^	C_15_H_18_O
2	6.045 ^b^	231.1281 ^c^	6.042 ^b^	231.1383 ^c^	C_15_H_18_O_2_
3	5.587 ^b^	321.1700 ^c^	5.581 ^b^	321.1699 ^c^	C_18_H_24_O_5_
4	5.473 ^b^	231.1379 ^c^	5.470 ^b^	231.1383 ^c^	C_15_H_18_O_2_
5	4.402 ^b^	247.1330 ^c^	4.383 ^b^	247.1334 ^c^	C_15_H_18_O_3_
6	4.761 ^b^	249.1487 ^c^	4.749 ^b^	249.1486 ^c^	C_15_H_20_O_3_
7	9.024 ^b^	329.2491 ^d^	9.040 ^b^	329.2487 ^d^	C_22_H_24_O_2_

## Data Availability

Not applicable.

## Sample Availability

Samples of the test compounds are available from the authors.
